# Multi-level predictors of being up-to-date with colorectal cancer screening

**DOI:** 10.1007/s10552-023-01723-w

**Published:** 2023-06-07

**Authors:** Kristen Hassmiller Lich, Sarah D. Mills, Tzy-Mey Kuo, Chris D. Baggett, Stephanie B. Wheeler

**Affiliations:** 1 Department of Health Policy and Management, Gillings School of Global Public Health, University of North Carolina at Chapel Hill, 1105E McGavran-Greenberg Hall, Chapel Hill, NC CB #7411 USA; 2 Department of Health Behavior, Gillings School of Global Public Health, University of North Carolina at Chapel Hill, Chapel Hill, NC USA; 3 Lineberger Comprehensive Cancer Center, University of North Carolina at Chapel Hill, Chapel Hill, NC USA; 4 Department of Epidemiology, Gillings School of Global Public Health, University of North Carolina at Chapel Hill, Chapel Hill, NC USA

**Keywords:** Colorectal cancer, Screening, Disparities

## Abstract

**Purpose:**

Assessing factors associated with being up-to-date with colorectal cancer (CRC) screening is important for identifying populations for which targeted interventions may be needed.

**Methods:**

This study used Medicare and private insurance claims data for residents of North Carolina to identify up-to-date status in the 10th year of continuous enrollment in the claims data and in available subsequent years. USPSTF guidelines were used to define up-to-date status for multiple recommended modalities. Area Health Resources Files provided geographic and health care service provider data at the county level. A generalized estimating equation logistic regression model was used to examine the association between individual- and county-level characteristics and being up-to-date with CRC screening.

**Results:**

From 2012–2016, 75% of the sample (*n* = 274,660) age 59–75 was up-to-date. We identified several individual- (e.g., sex, age, insurance type, recent visit with a primary care provider, distance to nearest endoscopy facility, insurance type) and county-level (e.g., percentage of residents with a high school education, without insurance, and unemployed) predictors of being up-to-date. For example, individuals had higher odds of being up-to-date if they were age 73–75 as compared to age 59 [OR: 1.12 (1.09, 1.15)], and if living in counties with more primary care physicians [OR: 1.03 (1.01, 1.06)].

**Conclusion:**

This study identified 12 individual- and county-level demographic characteristics related to being up-to-date with screening to inform how interventions may optimally be targeted.

Colorectal cancer (CRC) is the third most common cause of cancer in the United States (US) [1]. It is the second leading cause of cancer death [1]. Routine CRC screening is the most effective way to reduce risk of, and death from, CRC. The US Preventive Services Task Force (USPSTF) recently updated their guidelines to recommend all individuals aged 45 to 75 years regularly receive CRC screening [2]. Prior to 2021, CRC screening was recommended for individuals starting at age 50 [3]. Receipt of routine CRC screening has been associated with a 67% reduction in the risk of death from CRC in the US [4]. CRC screening can find cancer early, when treatment is most effective [5]. It can also identify precancerous polyps so they may be removed before they become cancerous [5]. There are several screening options recommended by the USPSTF [3]. The most commonly used screening modality is colonoscopy. An individual is considered up-to-date with CRC screening if they have a colonoscopy at least once every 10 years [3, 6]. The next most commonly used screening modality are stool tests, which include the fecal immunochemical test (FIT), guaiac-based fecal occult blood test (FOBT), and stool DNA test [3, 6]. According to current guidelines, FIT and FOBT are recommended annually and stool DNA testing is recommended every one to three years [3, 6]. CT colonography and flexible sigmoidoscopy are recommended every five years, though these modalities are far less commonly used [3, 6].

Although CRC screening rates have increased over the past two decades, screening rates remain suboptimal. According to data from the Behavioral Risk Factor Surveillance System in 2018, 68.8% of individuals who were age-eligible (50–75 years) reported being up-to-date with CRC screening in the US, up from 65.5% in 2012 [7, 8]. Average self-reported rates of CRC screening in 2018 are close to the *Healthy People* goal that 70.5% of age-eligible individuals be up-to-date with CRC screening by 2020 [8, 9]. However, there is concern that the percentage of individuals who are up-to-date with CRC screening is actually lower than rates documented because these rates are based on participant self-report, which research finds is prone to over-estimation due to poor recall, social desirability bias, and limited knowledge about CRC screening guidelines [10].

In addition, demographic and geographic disparities in CRC screening persist [7, 8, 11–13]. Only half of those aged 50–54 years are up-to-date with CRC screening as compared to 79.2% of those aged 65–75 years [8]. Also, racial/ethnic minorities and individuals with lower socioeconomic status, no health insurance, and no regular healthcare provider have lower rates of CRC screening as compared to those who are non-Hispanic White, have a higher socioeconomic status, health insurance, and a regular healthcare provider, respectively [7, 8, 11–15]. Only 36% of individuals without a regular healthcare provider were up-to-date with CRC screening in 2018 [8]. Studies also have found CRC screening rates vary according to community-level demographic characteristics and by state [8, 13]. Screening rates are lower in nonmetropolitan as compared to metropolitan areas and in counties with lower, as compared to higher, socioeconomic status [8, 12]. Individual- and community-level disparities in screening are a public health problem because they contribute to demographic disparities in CRC-related morbidity and mortality.

Assessing characteristics associated with being up-to-date with CRC screening is important for identifying populations for which targeted interventions may be needed. Due to the different frequency of CRC screenings recommended across screening modalities and the use of these tests for both screening and diagnostic procedures, assessing an individual’s up-to-date status is complex. Joseph et al. [7, 8] have used national, self-reported cross-sectional data from the Behavioral Risk Factor Surveillance System Survey to determine whether or not an individual was up-to-date with CRC screening in the US, but the authors noted their estimates may be inaccurate because participants did not specify if testing was done for CRC screening, to address symptoms, or for diagnosis [7]. In addition to distinguishing screening from other procedures, accurately assessing whether or not an individual is up-to-date with CRC screening requires knowing an individual’s screening history for at least 10 years. For example, if no screening is observed over a nine-year age-eligible window, we cannot determine whether that person was up-to-date or not for the entire period (i.e., whether or not they had a colonoscopy in the year prior). Despite this, state-level and national studies often use data that are cross-sectional or longitudinal data spanning less than a decade [7, 8, 11–13]. Another approach is to estimate screening compliance in individuals turning 50, among whom fewer years of continuous enrollment is required to ascertain up-to-date status [11, 13]. This method, however, precludes assessment of up-to-date status at older ages and assumes screening did not occur much before the age at which screening was first recommended.

To address these gaps, this study leveraged 14 continuous years of linked public and private insurance claims data to examine multi-level predictors of being up-to-date with CRC screening among individuals age-eligible in North Carolina and to assess the type of screening modality used among those who are up-to-date. The longitudinal insurance claims data allows for a comprehensive assessment of CRC screening history and up-to-date determination. We assessed CRC screening by modality to examine whether there has been a change in the type of modality used for CRC screening over time. Recognizing the time limited coverage offered by FIT, FOBT, and stool DNA tests, we also assessed repeated use of FIT, FOBT, or stool DNA tests to assess the percentage of individuals who are up-to-date among these individuals for whom stool test screening is intended to be completed annually.

## Methods

### Data, population, and inclusion/exclusion criteria

This study uses public Medicare and private health insurance claims data from 2003 to 2016 for residents of North Carolina. Direct identifiers were used to link individuals across public and private insurance claims datasets. All healthcare encounters that require a billing code are included in insurance claims data, including information about CRC screening. Insurance claims data have previously been used to assess CRC screening patterns [12, 13, 16].

North Carolina residents eligible for CRC screening with available health insurance claims data formed the primary analytic sample, which was then linked to Area Health Resource Files (AHRF) county-level contextual data. The AHRF data were used to gather geographic and health care service provider data at the county level [17]. AHRF data are released annually by the Bureau of Health Workforce and include data on population characteristics, health care professionals, health care facilities, hospital utilization and expenditures, and other health-related data at county, state, and national levels [17]. AHRF data were linked to insurance claims data using members’ county of residence. The insurance claims data and AHRF data were previously linked by analysts within the University of North Carolina (UNC) Lineberger Cancer Information and Population Health Resource, which holds data use agreements approving the ongoing linkage of these access-restricted data and their use for population health research [18]. The final analytic dataset was created for this study to incorporate the relevant inclusion/exclusion criteria. The research team obtained approval to conduct the study from the UNC Institutional Review Board.

**Fig. 1:**
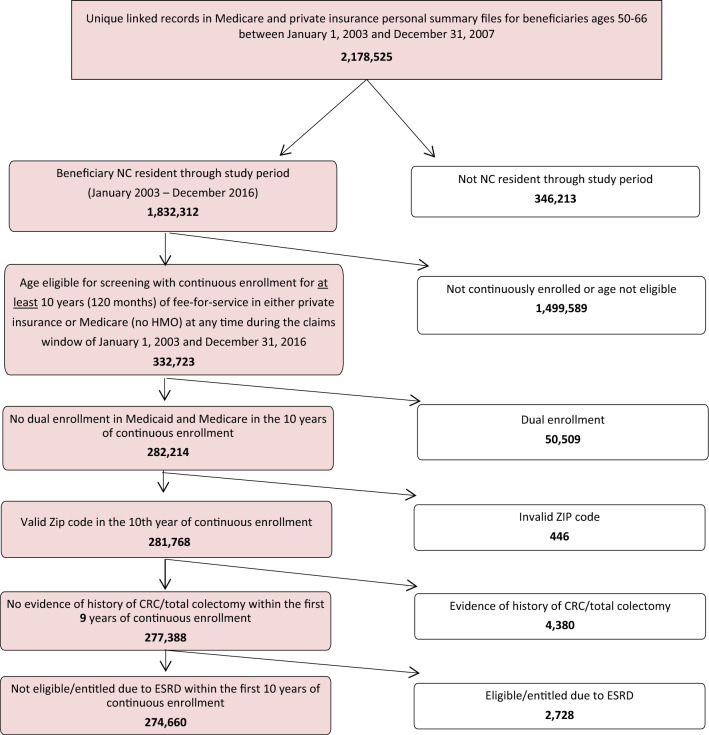
Inclusion and exclusion criteria applied to generate the analytic sample**.** *Notes.* NC = North Carolina. CRC = colorectal cancer. ESRD = end-stage renal disease. HMO = Health Maintenance Organization. Data was included in regression analyses beyond 10 years as long as an individual did not meet one of the following censoring criteria: 1) have an invalid zip code in that year, 2) have a diagnosis of CRC/total colectomy in that year, 3) have a diagnosis of ESRD in that year, 4) turn 76 or older in that year, 5) are enrolled in a HMO that year, or 6) with dual enrollment that year

The study population includes residents of North Carolina enrolled in Parts A and B of Medicare and/or with private insurance plans. Data from the private insurance plans included in this study cover a large percentage of the total private market in North Carolina. We began by selecting anyone enrolled between the ages of 50 and 66 between January 1, 2003 and December 31, 2007. Figure 1 presents inclusion/exclusion criteria. To ensure a comprehensive assessment of CRC screening, the study population was further limited to individuals with at least 10 years (120 months) of continuous enrollment (linked across payers) anytime during the claims window of Jan 1, 2003 through Dec 31, 2016. We required that individuals be continuously enrolled for at least 10 years to develop a sample for whom we know with certainty their up-to-date status in the 10th year or in subsequent years. The population also was age-eligible for CRC screening, according to the USPSTF guidelines, in at least their 10th year of continuous enrollment during the study period [3]. Our method for sample selection ensured that the youngest individuals in the sample would be newly age-eligible (50 years old) for CRC screening at the start of the study period in 2003. The oldest individuals in the sample would still be age-eligible (75 years old) for screening in 2012 after ten years of continuous enrollment.

Individuals were excluded from the dataset if they were dually enrolled in Medicare and Medicaid during the 10-year continuous enrollment period because Medicaid data were not available for the entire study period. In addition, participants’ zip code was needed to define a variable in the analysis, so individuals were excluded if they did not have a valid zip code in the 10th year of continuous enrollment. Individuals also were excluded if they had a total colectomy or history of CRC within the initial nine years of the continuous 10-year enrollment period to ensure the claims data reflected screening as opposed to CRC surveillance or other procedures. Finally, individuals were excluded if they had end-stage renal disease because CRC screening is not recommended for those with this terminal disease. A history of CRC, total colectomy, or end-stage renal disease were determined based on diagnosis and procedure codes in the claims data.

### Dependent variable

The primary study outcome was a dichotomous variable indicating whether an individual was up-to-date with CRC screening (1 = up-to-date; 0 = not up-to-date). To ensure a complete assessment of CRC screening, up-to-date status was assessed in the 10th year of continuous enrollment in the insurance claims data and in any subsequent years of continuous enrollment for which an individual is age-eligible (i.e., up to age 75). We first assessed screening when an individual became age-eligible at 50 years old, so the earliest assessment of up-to-date status occurred ten years later at age 59. Consistent with USPSTF guidelines during the study period, a person was considered up-to-date with CRC screening if they had a colonoscopy within the past 10 years, flexible sigmoidoscopy within the past 5 years, CT colonography within the past 5 years, or a stool DNA, FIT, or gFOBT within the past year [3]. Screening services were identified in the claims data using the International Classification of Diseases, 9th and 10th Editions, Clinical Modification, Current Procedural Terminology, or Healthcare Common Procedure Coding System codes (Appendix Table 1).

*Screening Modality.* In a given year, individuals may be up-to-date with CRC screening based on their screening conducted in that year or in prior years. For example, an individual who received a colonoscopy two years ago and received no screening in the current year would be considered up-to-date with screening via colonoscopy in the current year. If an individual was up-to-date with more than one screening modality, we noted all modalities used. For example, if an individual receives a FIT and a colonoscopy in the same year and that individual goes on to receive no additional screening in the following year, they will be coded as being up-to-date with both modalities in the first year and with colonoscopy in the second year.

### Independent variables

Independent variables at the individual level included sex (male, female), type of insurance at year of assessment of up-to-date status (private with or without Medicare, Medicare only), age at year of assessment of up-to-date status (59, 60–64, 65–69, 70–72, 73–75), year of assessment (i.e., the 10th or higher year of continuous enrollment: 2012, 2013, 2014, 2015, 2016), distance from patient zip code centroid to nearest endoscopy facility (0–5 miles, 5–10 miles, 10–15 miles, 15–20 miles, 20–25 miles, 25 or more miles), number of medical comorbidities (0, 1, 2, 3 +), and recent primary care visit (yes, no). The number of medical comorbidities was determined by summing each of the 17 medical conditions an individual had that is evaluated in the Charlson Comorbidity Index, a widely used measure of comorbidities based on an assessment of medical conditions and their severity [19, 20]. We used the number of medical comorbidities instead of the Charlson Comorbidity Index because a count of comorbidities can be more easily interpreted that the Index. Assessment of primary care visits and medical comorbidities were based on claims data for the 12 months prior to the assessment of up-to-date status.

Independent variables at the county-level included the percentage of residents with a primary care physician, percentage of residents with less than a high school education, unemployment rate, percentage of residents who are uninsured, and percentage of residents who are non-White from the AHRF data. The primary care physician variable was dichotomized to reflect individuals who lived in counties above and below the median percentage of residents with a primary care physician in the state. All other county-level variables were quartiled at the state-level.

### Statistical analyses

Descriptive statistics are presented for all persons who had at least 10 years of continuous enrollment in the insurance claims data. At the 10th or higher year of continuous enrollment for which they remained age-eligible for routine CRC screening, we assessed the percentage of individuals who were up-to-date with screening in the total sample and across individual- and county-level demographic characteristics. In each year from 2012–2016, we also examined the percentage of CRC screenings by modality among individuals who were eligible for screening, continuously enrolled in the dataset for ten or more years (so up-to-date status can be verified), and up-to-date with screening in that calendar year. Among individuals who were up-to-date using a FIT, FOBT, or stool DNA test in their 10th year of continuous enrollment and were eligible for CRC screening in the subsequent four years, we examined the percentage of all future years in which they had a FIT, FOBT, or stool DNA test.

A three-level generalized estimating equation (GEE) logistic regression model was used to examine the association between individual- and county-level demographic characteristics and being up-to-date with CRC screening. Because up-to-date status was assessed in the 10th year and every following year of age-eligible continuous enrollment, an individual may have contributed more than one person-observation to the analytic sample. Accordingly, the three-level GEE model controlled for potential repeated measurement of individuals as well as clustering at individual and county levels. Analyses were conducted from May 2021 to March 2022.

## Results

### Descriptive statistics

The sample included 274,660 unique North Carolinians who were continuously enrolled in the insurance claims dataset for at least 10 years when they were age-eligible for CRC screening, and thus contributed at least one year to the analysis. Among these individuals, some contributed more than one year to the analysis. Specifically, among individuals with 10 years of continuous enrollment, 157,213 (57.2%), 95,512 (34.8%), 69,103 (25.2%), and 47,484 (17.3%) contributed 1, 2, 3, and 4 additional observations, respectively. Therefore, 17.3% of the of the sample contributed an up-to-date observation in all five years (10, 11, 12, 13, and 14 years of continuous enrollment), while the remaining 82.7% contributed between 1 and 4 years. Table 1 presents personal-level characteristics by up-to-date status, assessed at the 10th year of continuous enrollment. Individuals in the 10th year of continuous enrollment were 59 to 75 years old. The sample was 46% male. The majority (76%) had a recent primary care visit and 68% were living within 10 miles of an endoscopy facility at the time of their up-to-date assessment. Fifty-nine percent of participants had 0 medical comorbidities (range: 0–10). Regarding county-level characteristics, 71% of the sample lived in a county with a primary care physician rate above the median in the state (4.8 physicians/10,000 persons). More than half (55%) of the sample lived in counties where 23% or more of the residents were non-white.

**Table 1:** Patient characteristics and percent up-to-date with colorectal cancer screening

Characteristic		Up-to-Date with CRC Screening		
		Total (*N* = 274,660)	Yes (*N* = 208,790)	No (*N* = 65,870)
Year of up-to-date assessment	2012	129,855 (47%)	98,968 (76%)	30,887 (24%)
	2013	38,781 (14%)	29,265 (75%)	9,516 (25%)
	2014	36,939 (13%)	27,865 (75%)	9,074 (25%)
	2015	37,691 (14%)	28,941 (77%)	8,750 (23%)
	2016	31,394 (11%)	23,751 (76%)	7,643 (24%)
Age group at year of UTD (years)	59	26,979 (10%)	20,538 (76%)	6,441 (24%)
	60–64	49,265 (18%)	36,901 (75%)	12,364 (25%)
	65–69	48,110 (18%)	36,394 (76%)	11,716 (24%)
	70–72	37,213 (14%)	28,997 (78%)	8,216 (22%)
	73–75	113,093 (41%)	85,960 (76%)	27,133 (24%)
Sex	Male	126,748 (46%)	92,642 (73%)	34,106 (27%)
	Female	147,912 (54%)	116,148 (79%)	31,764 (21%)
Primary care visit in the prior year	Yes	209,408 (76%)	168,930 (81%)	40,478 (19%)
	No	65,252 (24%)	39,860 (61%)	25,392 (39%)
Number of primary care physician visits in the prior year	Mean (SD)	4.13 (4.99)	4.49 (5.09)	3.01 (4.45)
Number of comorbidities	0	163,394 (59%)	121,309 (74%)	42,085 (26%)
	1	68,026 (25%)	53,645 (79%)	14,381 (21%)
	2	25,244 (9%)	19,898 (79%)	5,346 (21%)
	3 +	17,996 (7%)	13,938 (77%)	4,058 (23%)
Distance to nearest endoscopy facility (miles)	< = 5	92,492 (34%)	71,502 (77%)	20,990 (23%)
	5–10	92,260 (34%)	70,239 (76%)	22,021 (24%)
	10–15	47,156 (17%)	35,689 (76%)	11,467 (24%)
	15–20	23,703 (9%)	17,380 (73%)	6,323 (27%)
	20–25	11,524 (4%)	8,469 (73%)	3,055 (27%)
	25 +	7,525 (3%)	5,511 (73%)	2,014 (27%)
Insurance	private insurance with/without Medicare	73,193 (27%)	57,565 (79%)	15,628 (21%)
	Medicare only	201,467 (73%)	151,225 (75%)	50,242 (25%)
Primary care physician/10,000 persons	Above median (> = 4.8/10,000)	196,042 (71%)	151,092 (77%)	44,950 (23%)
	Below median (< 4.8/10,000)	78,618 (29%)	57,698 (73%)	20,920 (27%)
% with less than a high school education	Low: < 13%	137,392 (50%)	107,230 (78%)	30,162 (22%)
	Low-Med: 13–17%	53,908 (20%)	40,953 (76%)	12,955 (24%)
	Med-High: 17–21%	50,610 (18%)	37,025 (73%)	13,585 (27%)
	High: > 21%	32,750 (12%)	23,582 (72%)	9,168 (28%)
Unemployment rate	Low: < 5%	103,516 (38%)	80,465 (78%)	23,051 (22%)
	Low-Med: 5–6%	76,638 (28%)	57,253 (75%)	19,385 (25%)
	Med-High: 6–7%	50,608 (18%)	38,635 (76%)	11,973 (24%)
	High: > 7%	43,898 (16%)	32,437 (74%)	11,461 (26%)
% without insurance	Low: < 13%	89,979 (33%)	70,444 (78%)	19,535 (22%)
	Low-Med: 13–14%	84,285 (31%)	63,814 (76%)	20,471 (24%)
	Med-High: 14–15%	64,568 (24%)	48,567 (75%)	16,001 (25%)
	High: > 15%	35,828 (13%)	25,965 (72%)	9,863 (28%)
% of residents who are non-White	Low: < 10%	37,419 (14%)	26,871 (72%)	10,548 (28%)
	Low-Med: 10–23%	85,658 (31%)	65,034 (76%)	20,624 (24%)
	Med-High: 23–38%	77,963 (28%)	60,140 (77%)	17,823 (23%)
	High: > 38%	73,620 (27%)	56,745 (77%)	16,875 (23%)

*CRC*  colorectal cancer. *SD* standard deviation. Only individuals first year of UTD assessment is included

### Demographic characteristics associated with being up-to-date with CRC screening

In 2012, 2013, 2014, 2015, and 2016, 76%, 75%, 75%, 77%, and 76% of the sample was up-to-date with CRC screening, respectively (Table 1). Overall, in the 10th year of continuous enrollment in the insurance claims data, a greater percentage of women (79%) were up-to-date with CRC screening as compared to men (73%). A greater percentage of individuals with private insurance (79%) were up-to-date as compared to those with Medicare only (75%). Also, 81% of individuals who had a primary care visit in the year prior to the assessment of up-to-date status were up-to-date, as compared to only 61% of those who had no primary care visit in the year prior to assessment. Overall, a greater percentage of individuals were up-to-date with CRC screening in counties with a higher percentage of residents with more than a high school education, higher employment rate, and greater percentage of residents with insurance. More individuals were up-to-date in counties with a higher percentage of non-White residents.

### Screening modalities

We further examined the screening modality with which an individual is up-to-date in each calendar year (Table 2). We limited this descriptive analysis to the subset of all individuals who were up-to-date in that calendar year. An individual may have been up-to-date with screening because of a screening completed in that calendar year or in prior years. The majority (81.3%) of years up-to-date were with colonoscopies and 16.6% of years up-to-date were with a FIT or FOBT. A minority (< 3%) of years up-to-date were due to a stool DNA test, sigmoidoscopy, or CT colonography. Among individuals who had a FIT, FOBT, or stool DNA test in their 10th year of continuous enrollment and were eligible for screening in the subsequent four years (i.e., excluding individuals who age-out or receive a colonoscopy and are no longer due for screening in this four-year period; *n* = 1,513), 39%, 20%, 11%, and 6% of individuals had 1, 2, 3, and 4 more stool-based tests in the next four years, respectively. Among individuals who were up-to-date with CRC screening by stool test and did not receive a follow-up colonoscopy, only 17.9% of the annual stool test screenings that would be recommended in subsequent years were actually received.

**Table 2:** Colorectal cancer screening by modality, 2012–2016

Calendar year	People Up-to-Date with Screening	Number of screenings	People Up-to-Date by Modality									
			Colonoscopy		FIT or FOBT		Stool_DNA		Sigmoidoscopy		CT_colonography	
	**N**	**N**	N	%	N	%	N	%	N	%	N	%
2012–2016	495,811	586,973	476,932	81.3	97,495	16.6	1025	0.2	10,406	1.8	1115	0.2
2012	98,968	119,721	94,757	79.1	22,441	18.7	0	0.0	2313	1.9	210	0.2
2013	106,337	127,285	102,426	80.5	22,341	17.6	0	0.0	2289	1.8	229	0.2
2014	92,272	109,038	88,933	81.6	17,956	16.5	14	0.0	1929	1.8	206	0.2
2015	99,147	116,096	95,607	82.4	18,070	15.6	238	0.2	1948	1.7	233	0.2
2016	99,087	114,833	95,209	82.9	16,687	14.5	773	0.7	1927	1.7	237	0.2

The percentage is based on the total colorectal cancer screenings in each year. A person could be up-to-date with multiple modalities in a single year. For the row that aggregates across years (2012–2016), all findings reflect person-years

### Generalized estimating equation model

In the adjusted GEE model (Table 3), individuals had higher odds of being up-to-date with CRC screening if they were female [Odds Ratio [OR]: 1.34 (1.32, 1.37)], had a recent primary care visit [OR: 1.31 (1.30, 1.33)], or had private insurance [OR: 1.30 (1.27, 1.33)]. Individuals also had higher odds of being up-to-date if they were older [ORs range: 1.07 (1.06, 1.09) -1.19 (1.16, 1.22)], had more comorbidities [ORs range: 1.14 (1.12, 1.15)–1.19 (1.17, 1.22)], and lived closer to, as compared to further away from, the nearest endoscopy facility [ORs range: 0.89 (0.86, 0.92)–0.94 (0.92, 0.97)]. The odds of being up-to date with CRC screening was also greater in later years of the study period [ORs range: 1.12 (1.10, 1.13)–1.14 (1.12, 1.15)].

**Table 3:** Predictors of being up-to-date with CRC screening

Characteristic		Odds Ratio	95% Confidence Interval		*p*-value
Sex	Female	1.34	1.32	1.37	< .0001
	Male	1	1	1	
Age at UTD assessment (years)	60–64	1.07	1.06	1.09	< .0001
	65–69	1.18	1.15	1.21	< .0001
	70–72	1.19	1.16	1.22	< .0001
	73–75	1.12	1.09	1.15	< .0001
	59	1	1	1	
Year at UTD assessment	2013	1.12	1.11	1.12	< .0001
	2014	1.12	1.10	1.13	< .0001
	2015	1.12	1.10	1.13	< .0001
	2016	1.14	1.12	1.15	< .0001
	2012	1	1	1	
Visit with primary care provider	Yes	1.31	1.30	1.33	< .0001
	No	1	1	1	
Comorbidities	1	1.14	1.12	1.15	< .0001
	2	1.17	1.15	1.19	< .0001
	3 +	1.19	1.17	1.22	< .0001
	0	1	1	1	
Distance to nearest endoscopy facility (miles)	5–10	0.94	0.92	0.96	< .0001
	10–15	0.94	0.92	0.97	< .0001
	15–20	0.88	0.86	0.91	< .0001
	20–25	0.91	0.87	0.95	< .0001
	> = 25	0.91	0.86	0.95	0.0004
	0–5	1	1	1	
Insurance type	private insurance with/without Medicare	1.30	1.27	1.33	< .0001
	Medicare only	1	1	1	
% with less than a high school education	Low-Med	0.96	0.93	0.99	0.003
	Med-High	0.87	0.84	0.89	< .0001
	High	0.83	0.80	0.86	< .0001
	Low	1	1	1	
Unemployment rate	Low-Med	0.96	0.94	0.98	0.0005
	Med-High	1.02	0.99	1.05	0.2048
	High	0.86	0.84	0.89	< .0001
	Low	1	1	1	
% without insurance	Low-Med	0.88	0.86	0.91	< .0001
	Med-High	0.92	0.89	0.94	< .0001
	High	0.93	0.90	0.96	< .0001
	Low	1	1	1	
% of residents who are non-White	Low-Med	1.17	1.14	1.20	< .0001
	Med-High	1.22	1.19	1.26	< .0001
	High	1.31	1.27	1.35	< .0001
	Low	1	1	1	
Presence of primary care physician/10,000 persons	Above median	1.03	1.01	1.06	0.0102
	Below median	1	1	1	

*UTD *Up-to-date

Regarding county-level characteristics, the odds of being up-to-date with CRC screening were higher for people who lived in counties with more primary care physicians [OR: 1.03 (1.01, 1.06)] and higher for people who lived in counties with a greater, as opposed to a lower, percentage of residents who are non-White [ORs range: 1.17 (1.14, 1.20)–1.31 (1.27, 1.35)]. In addition, the odds of being up-to-date with CRC screening were typically higher for people who lived in counties with a lower percentage of residents with less than a high school education [ORs range: 0.83 (0.80, 0.86)–0.96 (0.93, 0.99)], a lower unemployment rate [ORs range: 0.86 (0.84, 0.89)–1.02 (0.99, 1.05)], and a lower percentage of residents without insurance [ORs range: 0.88 (0.86, 0.91)–0.93 (0.90, 0.96), Table 3].

**Fig. 2:**
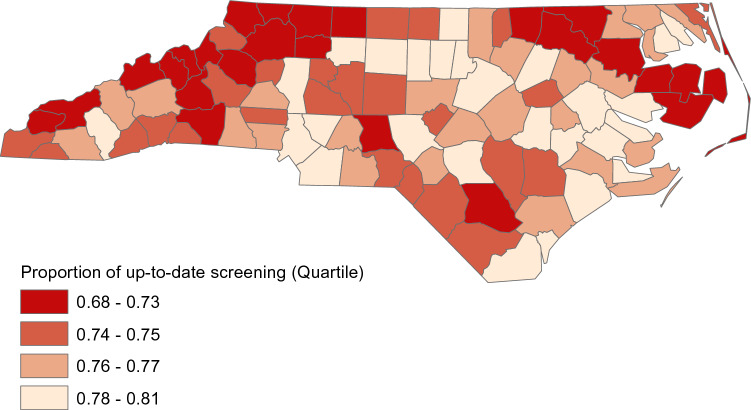
Model Predicted Proportion of Up-to-date Colorectal Cancer Screening in North Carolina Counties

Figure 2 presents the model predicted proportion of individuals up-to-date with CRC screening in each county in North Carolina. A greater percentage of individuals living in counties in the central and eastern regions of the state were up-to-date with CRC screening.

## Discussion

This study utilized Medicare and private insurance claims data spanning more than a decade to examine individual- and county-level predictors of being up-to-date with CRC screening in a sample of age-eligible North Carolina residents. Prior research indicates that the percentage of individuals up-to-date with CRC screening has increased, but remains suboptimal. In the present study, rates of being up-to-date with CRC screening were stable from 2012–2016, at 75%–77%. The higher rates of being up-to-date with CRC screening in this study, compared to studies of all adults age-eligible for CRC screening, may be due to the large percentage (55%) of older adults (70–75 years) in the study sample. We limited assessment of whether or not an individual was up-to-date with screening to those 59 years and older because we required at least 10 years of continuous enrollment after age 50 (when individuals become eligible for screening) to ensure an accurate assessment of whether or not an individual was up-to-date. Also, individuals with Medicaid or who were uninsured during the study period were excluded, which likely increased estimates of up-to-datedness. Among those age-eligible for screening, it is well established that older adults are more likely to be up-to-date with CRC screening as compared to those who are younger [7, 8]. In addition, screening rates among individuals with Medicaid or who are uninsured are typically lower than those with Medicare or private insurance [8, 21, 22]. Despite relatively high rates of being up-to-date with CRC screening overall, there was significant heterogeneity in the percentage of individuals who were up-to-date across participant demographic characteristics. The present study identified twelve individual- and county-level demographic characteristics related to being up-to-date with screening that can inform how interventions to increase CRC screening rates may be optimally targeted.

Overall, as expected, findings from the present study are largely consistent with prior research that has documented individual- and county-level demographic disparities in CRC screening in North Carolina and in other states and localities in the US. [7, 8, 11–13, 23] For example, prior studies of individuals with public insurance who were newly age-eligible for CRC screening also found women and those with a recent primary care visit had a higher odds of receiving CRC screening than men or individuals without a recent primary care visit, respectively [12, 13]. However, these prior studies found more pronounced relationships between some individual-level demographic characteristics and CRC screening than what was found in the present study. [12, 13] For example, in a prior study a recent primary care visit was associated with 2.5 (2.4, 2.6) higher odds of receipt of CRC screening among individuals newly age-eligible for CRC screening as compared to a 1.31 (1.30, 1.33) higher odds found in the present study [12]. In another study, women (as compared to men) had 2.2 (2.0, 2.3) higher odds of CRC screening as compared to 1.3 (1.3, 1.7) higher odds found in the present study[13]. Individuals newly age-eligible for screening may have more pronounced demographic disparities in screening that lessen when evaluating screening among the total age-eligible population as more individuals become aware they are eligible.

Relationships between county-level demographic characteristics and CRC screening found in the present study were less consistent with prior research. In the present study, several county-level characteristics based on insurance status, education, primary care access, and racial/ethnic composition were significantly associated with being up-to-date with CRC screening. A prior study of individuals newly age-eligible for screening did not find significant associations between these county-level variables and receipt of screening [13]. This may be due to differences in the age of the samples across studies. In addition, the present study evaluated whether or not an individual was up-to-date in 2012 to 2016, while the prior study evaluated receipt of screening in 2003 to 2008 [13]. Findings from the present study may be more generalizable to the total population currently age-eligible for screening. Also, in the present study, individuals living in counties with a higher proportion of non-White residents were more likely to be up-to-date with screening. This is inconsistent with a prior study using Medicare and Medicaid data that found no significant relationship between the proportion of non-White residents in a county and receipt of CRC screening.[13]. In the present study, the proportion of the study sample living in urban areas was higher in counties that had a higher percentage of non-White residents. This may explain the improved screening in counties with a greater proportion of non-White residents because access to screening may be better in urban as compared to rural areas [24]. Studies should also examine if there are particular racial/ethnic minority groups with relatively high screening rates that may be driving this effect in the present study. Rates of being up-to-date with CRC screening were also generally lower in the northwest and northeast regions of the state. Counties in these regions tend to be rural and have lower median household incomes [25].

Targeted public health interventions among individuals with lower screening rates such as those who are younger, have not had a recent primary care visit, and live in counties with lower socioeconomic status are needed to reduce demographic disparities in screening in the state. Partnerships between public health and primary care clinics focused on implementing evidence-based interventions to increase CRC screening have been found to be successful in lower socioeconomic areas in the US [8]. Evidence-based interventions to increase CRC screening include sending patient and provider reminders about CRC screening, pairing screening with other preventive services, and reducing structural barriers to screening (e.g., expanding screening hours, reducing transportation barriers, providing screening navigation support) [26, 27]. Additionally, the Community Preventive Services Task Force finds that *multicomponent* interventions that combine several strategies are most effective at increasing screening rates [27]. Educating individuals about the benefits of screening at early ages may also help reduce age-based disparities in screening [8].

Consistent with prior research, in the present study the majority (81.3%) of years up-to-date were due to having received a colonoscopy in the past 10 years. Fewer (16.6%) years up-to-date were due to having received a FIT or FOBT. Receipt of stool DNA, sigmoidoscopy, and CT colonography was not common. From 2012 to 2016, the percentage up-to-date via colonoscopy increased, while the percentage that were up-to-date through FIT or FOBT declined. Although colonoscopy is the most commonly used screening modality, other research suggests that there has been an increase in the use of stool tests as these tests have become more available, perhaps further spurred by the benefits of at-home care during COVID-19 [23, 28–30]. In the present study the percentage of screenings conducted using stool DNA increased modestly from 2015 to 2016, but there was either no change or a decline in receipt of sigmoidoscopy and CT colonography over the study period. Notably, in a subsample of individuals who received a FIT or FOBT, remained age-eligible for screening for four additional years, and did not receive a colonoscopy (i.e., a group in which we expect four additional FIT or FOBTs), few (6%) received four FIT or FOBTs in the subsequent four years. This raises concern that individuals receiving FITs or FOBTs may be at higher risk for not being up-to-date with screening over time.

Akram et al. [31] and others[32] recommend healthcare systems replace provision of FOBTs with FITs to improve screening rates among those using stool tests because FITs require 1 sample, as opposed to 3, and there are no dietary restrictions, increasing the likelihood of test completion. Research finds that *mailed* outreach of stool tests are more effective than clinic-based offers of screening [33]. This may be because mailed outreach can increase reach of the intervention by connecting screening to individuals with barriers to in-person clinical care [33]. The mailed test also acts as a reminder itself and can provide educational information about screening [33]. Multitarget stool DNA tests, under the brand name Cologuard, are another recommended alternative to FIT, with benefits of increased accuracy and ability to test every one to three years [6]. It is important that future research continue to study evolving modality use and the percentage of individuals up-to-date by screening modality over time. The percentage of individuals up-to-date using stool tests may increase as these tests become more commonly used or if FITs/multitarget stool DNA tests are more often offered by healthcare providers. With screening tests that require more frequent use, attention should be given to potential differences in receipt of screening/testing (incidence) and rates of up-to-datedness over time.

There are limitations to the present study. Private and Medicare insurance claims data were used to assess receipt of CRC screening. Individuals with Medicaid were excluded from the analysis because their insurance claims data was not available over the entire study period, which may bias the sample in the present study toward older and higher-income individuals. The study sample also excluded uninsured individuals and a small portion of the market with private insurance. Therefore, results from the study may not be generalizable to the total population age-eligible for CRC screening in North Carolina or to other populations. Comparison of the age and sex composition of the sample in the present study to the total population age-eligible for CRC screening in North Carolina over the same study period suggests the present study sample was more representative of older adults [34]. Also, this study sample of older adults had a relatively low number of comorbidities [35, 36]. In North Carolina, an estimated 83% of adults aged 65 and older have at least one chronic disease [37]. We also used the more conservative one-year screening interval recommended by the US Preventive Services Task Force at the time of the study to determine whether or not an individual was up-to-date when using stool DNA tests. This may have biased the results to indicate that a greater percentage of people using stool DNA were not up-to-date; however, a very small percentage (0.2%) of the sample used stool DNA tests in the study.

Overall, the present study found relatively high rates of being up-to-date with CRC screening among Medicare and privately insured persons in North Carolina. Nonetheless, rates varied significantly according to demographic characteristics at individual- and county-levels, raising concern that differences in screening may lead to demographic and geographic disparities in CRC-related morbidity and mortality. To increase CRC screening rates and reduce disparities, interventions should target populations identified in the present study with lower screening rates, such as those who have who have not had a recent primary care visit or live in counties with lower socioeconomic status.

## Data Availability

The datasets analyzed for the current study are not publicly available due to data use restrictions with the insurance claims data.

## Appendix

**Appendix Table 1:** Billing codes indicating colorectal cancer screening procedures

Category	Codes
**Screening test modality**	
Fecal occult blood test or Fecal Immunochemical Test	CPT: 82,270 (FOBT), 82,271, 82,272, 82,273, 82,274 (FIT) HCPCS: G0328 (FIT), G0107 (FOBT)
Colonoscopy	CPT: 44,388, 44,389, 44,390, 44,391, 44,392, 44,393, 44,394, 44,397, 44,401, 44,402, 44,403, 44,404, 44,405, 44,406, 44,407, 44,408, 45,355, 45,378, 45,379, 45,380, 45,381, 45,382, 45,383, 45,384, 45,385, 45,386, 45,387, 45,388, 45,389, 45,390, 45,391, 45,392, 45,393, 45,398 HCPCS: G0105, G0121 ICD-9 Procedure: 45.21, 45.22, 45.23, 45.25, 45.41, 45.42, 45.43, 48.36 ICD-10 Procedure: 0DJD4ZZ, 0DJD8ZZ, 0DBE8ZX, 0DBE0ZZ, 0DBE3ZZ, 0DBE7ZZ, 0DBE4ZZ, 0DBE8ZZ, 0D5E4ZZ, 0D5E8ZZ, 0DBP4ZZ, 0DBP8ZZ
Flexible Sigmoidoscopy	CPT: 45,300, 45,303, 45,305, 45,307, 45,308, 45,309, 45,315, 45,317, 45,320, 45,321, 45,327, 45,330, 45,331, 45,332, 45,333, 45,334, 45,335, 45,337, 45,338, 45,339, 45,340, 45,341, 45,342, 45,345, 45,346, 45,347, 45,349, 45,350 HCPCS: G0104 ICD-9 Procedure: 45.24, 48.21, 48.22, 48.23, 48.24 ICD-10 Procedure: 0DJD8ZZ, 0DJD4ZZ, 0DBP8ZX
Stool DNA Test	CPT/HCPCS: 81,528, G0464
CT Colonography	CPT: 74,263, 74,261, 74,262, 0066 T, 0067 T

*FOBT* Fecal occult blood test, *FIT* Fecal Immunochemical Test, *ICD-9-CM* International Classification of Diseases, *9th Edition* Clinical Modification, *ICD-10-CM* International Classification of Diseases, *10th Edition* Clinical Modification, *CPT* Current Procedural Terminology, *HCPCHS* Healthcare Common Procedure Coding System
